# Skin toxicity and quality of life during treatment with panitumumab for *RAS* wild-type metastatic colorectal carcinoma: results from three randomised clinical trials

**DOI:** 10.1007/s11136-016-1288-4

**Published:** 2016-04-15

**Authors:** Reija Koukakis, Francesca Gatta, Guy Hechmati, Salvatore Siena

**Affiliations:** 1 Biostatistics, Amgen Ltd, 1 Uxbridge Business Park, Sanderson Road, Uxbridge, Middlesex, UB8 1DH UK; 2 Global Health Economics, Amgen (Europe) GmbH, Zug, Switzerland; 3 Niguarda Cancer Center, Grande Ospedale Metropolitano Niguarda, Milan, Italy; 4 Università degli Studi di Milano, Milan, Italy

**Keywords:** Quality of life, Colorectal cancer, Panitumumab, Epidermal growth factor receptor inhibitors, Skin toxicity

## Abstract

**Purpose:**

Epidermal growth factor receptor inhibitors such as panitumumab are associated with characteristic skin toxicities. We summarise data from three panitumumab clinical trials to investigate the potential impact of skin toxicity on quality of life (QoL) in patients with metastatic colorectal cancer (mCRC).

**Methods:**

The studies were randomised, open-label trials comparing standard treatment (first-line FOLFOX4 [*n* = 456], second-line FOLFIRI [*n* = 381], or best supportive care [*n* = 114]) with or without panitumumab in adults with *KRAS/NRAS* (*RAS*) wild-type mCRC. QoL was assessed using the EuroQoL 5-domain health state index (HSI) and overall health rating (OHR) measures. Impact of skin toxicity on changes in QoL scores was estimated using a linear mixed-effects model. Worst skin toxicity was defined in separate models as a subgroup variable or as a measure over time.

**Results:**

Regardless of analysis method, there were no statistically significant differences between the panitumumab and comparator arms in any of the studies in terms of change in HSI or OHR scores. There were no statistically significant differences in QoL outcomes between patients with worst skin toxicity grade <3 and those with grade ≥3. In addition, there were no statistically significant differences between the panitumumab and comparator arms in subgroups of patients with worst skin toxicity of grade <3 and ≥3.

**Conclusions:**

Addition of panitumumab to chemotherapy in *RAS* wild-type mCRC has no statistically significant negative effect on overall QoL, despite skin toxicity. Skin toxicity of worst grade ≥3 appeared to have similar impact on QoL as skin toxicity of grade <3.

**Electronic supplementary material:**

The online version of this article (doi:10.1007/s11136-016-1288-4) contains supplementary material, which is available to authorized users.

## Introduction

The epidermal growth factor receptor (EGFR) is a receptor tyrosine kinase that plays a key role in the development and progression of some tumours, particularly colorectal carcinoma [1]. It is therefore an attractive target for anticancer therapies. Panitumumab is a fully human monoclonal antibody that targets EGFR [2]. Studies have shown that panitumumab can significantly improve progression-free survival (PFS) across several lines of treatment in patients with metastatic colorectal cancer (mCRC) whose tumours are wild type (WT) for the *RAS* oncogene (i.e. no detectable mutations in both the *KRAS* and *NRAS* genes) [3–6]. In particular, addition of panitumumab to chemotherapy with leucovorin, 5-fluorouracil, and oxaliplatin (FOLFOX, as either the standard FOLFOX4 or the more intensive FOLFOX6 regimen) in first-line treatment has been shown to improve overall survival (OS) versus FOLFOX alone [3] and versus FOLFOX plus bevacizumab [7] in patients with WT *RAS* or *KRAS* mCRC. As a result, panitumumab was licensed for the treatment of patients with *RAS* WT mCRC. The licensed indications in Europe are first-line therapy in combination with FOLFOX or FOLFIRI (leucovorin, 5-fluorouracil, and irinotecan), as second-line therapy in combination with FOLFIRI, and as monotherapy after failure of multiple chemotherapy regimens [8].

Adverse events during cancer treatment can have a negative effect on quality of life (QoL) [9, 10], and optimal therapy, therefore, involves a balance between efficacy and safety [11]. Dermatological toxicities such as papulopustular rash (acneiform eruption), erythema, and skin fissures are common side effects of targeted cancer agents such as EGFR inhibitors [12], as EGFR is involved in the normal development and physiology of the epidermis. It has been reported that emergence of skin toxicity may be a surrogate clinical marker for efficacy of EGFR inhibitors in mCRC, although this remains controversial, with few prospective studies. Studies have also investigated the link between QoL and outcomes in colorectal cancer, showing that baseline QoL is an independent predictor for survival [13]. In patients receiving panitumumab in combination with FOLFOX, the occurrence of skin toxicity has been correlated with improved survival outcomes in patients with mCRC [14], but this association is not clear and may be related to the longer duration of treatment in patients responding to panitumumab.

As part of three clinical trials of different lines of treatment with panitumumab in patients with mCRC, QoL data were collected as pre-specified tertiary endpoints: the 20050203 (‘PRIME’; NCT00364013) study in first-line treatment of mCRC [15]; the 20050181 (‘181’; NCT00339183) study in second-line treatment [16]; and the 20020408 (‘408’; NCT00113763) study in third- or fourth-line treatment [17]. Given that skin toxicity is a common side effect of panitumumab, we summarise QoL data from patients with *RAS* WT mCRC in those three studies to investigate a potential relationship between skin toxicity and QoL in patients receiving panitumumab.

## Methods

### Study designs and patients

Full details of the study design and inclusion criteria for the three included studies have been published previously [15, 17, 18]. All three studies were randomised, open-label phase III trials comparing a standard treatment regimen (PRIME, first-line FOLFOX4; 181, second-line FOLFIRI; 408, best supportive care [BSC]) with or without panitumumab. Eligible patients in each study were aged ≥18 years and had an Eastern Cooperative Oncology Group performance status of 0−2. In all three studies the panitumumab dose was 6.0 mg/kg every 2 weeks, and PFS was a primary endpoint. OS was a primary endpoint in the 181 study and a secondary endpoint in the other two studies, with other secondary endpoints in all three studies including objective tumour response and safety. The present analyses use data from the subset of patients with *RAS* WT mCRC from these three studies [3–5].

The protocols of all three studies were approved by the ethics committees at participating sites and adhered to all ethical guidelines, and all patients signed informed consent before any study-related procedures were performed.

### Skin toxicity

Adverse events were collected throughout treatment and safety follow-up in all three studies and graded according to National Cancer Institute’s Common Toxicity Criteria (version 3.0) [19], with the exception of panitumumab-related skin toxicities, which were graded using a modified version of the CTC version 3.0. Severity of adverse events was rated on a five-point scale: 1 = mild; 2 = moderate; 3 = severe; 4 = life threatening or disabling; and 5 = death.

### QoL endpoints and analyses

QoL was assessed as a pre-specified tertiary endpoint during each study, using the EuroQoL 5-domain (EQ-5D) health state index (HSI) and overall health rating (OHR) measures. HSI scores range from −0.594 to 1.0 (higher scores represent better health, with 1.0 equivalent to perfect health), while OHR comprises a 0−100 visual analogue scale, with 0 representing ‘Worst imaginable health state’ and 100 representing ‘Best imaginable health state’. QoL was assessed ≤7 days before randomisation and every 4 weeks until disease progression, with a final assessment at a safety follow-up visit. For all analyses, minimally important differences (MIDs) were defined as 0.08 for HSI and 7 for OHR [20]. A descriptive analysis of the distribution of worst skin toxicity grades in patients with a decrease in HSI or OHR exceeding the MID was also performed.

Primary and secondary QoL analyses were conducted on the *RAS* WT patient-reported outcome (PRO) patient cohort, defined as the subsets of *RAS* WT patients in each intent-to-treat analysis set who received at least one dose of study medication, and had a baseline QoL assessment and at least one post-baseline QoL assessment. This is an exploratory analysis without type-I error rate control, and all *p* values are descriptive.

### Primary analysis

The impact of worst skin toxicity grade on the changes from baseline to discontinuation of treatment in QoL scores was estimated using a linear mixed-effects model for repeated measures [21]. The mixed-effects model was used for all regression analyses and adjusted for treatment, visit, worst skin toxicity grade, and baseline HSI scores (termed as fixed effects), with intercept and visit as random effects. Worst skin toxicity grade was defined in two different ways in the linear mixed-effect models. In definition 1, between-treatment differences were assessed adjusting for fixed effects terms and significant covariate-by-covariate interactions at the 5 % level. Differences by worst skin toxicity level (grade <3 vs. ≥3) were evaluated adjusting for fixed effects terms and significant covariate-by-covariate interactions at the 5 % level. Between-treatment differences for each worst skin toxicity level (grade <3 and ≥3) were assessed using a full model with interactions included. In definition 2, worst skin toxicity was included as a measure over time (i.e. worst skin toxicity grade was assigned to each QoL assessment visit). Covariate-by-covariate interactions were assessed and retained in the model if statistically significant at the 5 % level, and between-treatment differences were assessed adjusting for fixed effect terms and significant interactions.

### Secondary analyses

In addition to the primary analysis, the distribution of worst skin toxicity grades by treatment arm for all grades was calculated in the subset of patients who had clinically meaningful decreases from baseline in QoL scores. As a sensitivity analysis, the mixed-effect model was repeated without skin toxicity as a covariate, and between-treatment differences were assessed adjusting for treatment, visit, and baseline HSI scores. Demography variables were summarised.

## Results

### Patients

The *RAS* WT PRO sets for PRIME included 232 patients who received panitumumab + FOLFOX4 and 224 who received FOLFOX4 alone; for the 181 study included 187 patients who received panitumumab + FOLFIRI and 194 who received FOLFIRI alone; and for the 408 study included 66 patients who received panitumumab + BSC and 48 who received BSC alone. Baseline demographics, disease characteristics, and QoL scores for patients in the three studies are shown in Table 1. Demographics and disease characteristics were generally similar across studies and between treatment groups, although median QoL scores were lower in the 408 study than in PRIME or 181.

**Table 1:** Baseline demographics and disease characteristics (*RAS* wild type, patient-reported outcome intent-to-treat population)

	PRIME		181		408	
	Panitumumab + FOLFOX4 (*n* = 232)	FOLFOX4 (*n* = 224)	Panitumumab + FOLFIRI (*n* = 187)	FOLFIRI (*n* = 194)	Panitumumab + BSC (*n* = 66)	BSC (*n* = 48)
Sex						
Male	153 (66)	145 (65)	120 (64)	126 (65)	42 (64)	32 (67)
Female	79 (34)	79 (35)	67 (36)	68 (35)	24 (36)	16 (33)
Age (years), median (range)	61.0 (27.0, 81.0)	61.0 (24.0, 82.0)	60.0 (28.0, 81.0)	60.5 (33.0, 85.0)	61.5 (29.0, 79.0)	62.5 (32.0, 81.0)
Age group, *n* (%) (years)						
<65	151 (65)	143 (64)	116 (62)	125 (64)	37 (56)	29 (60)
≥65	81 (35)	81 (36)	71 (38)	69 (36)	29 (44)	19 (40)
<75	213 (92)	201 (90)	178 (95)	182 (94)	57 (86)	42 (88)
≥75	19 (8)	23 (10)	9 (5)	12 (6)	9 (14)	6 (13)
ECOG performance score, *n* (%)						
0 or 1	221 (95)	209 (93)	180 (96)	184 (95)	60 (91)	41 (85)
2	11 (5)	15 (7)	7 (4)	10 (5)	6 (9)	7 (15)
Primary tumour diagnosis, *n* (%)						
Colon	151 (65)	148 (66)	107 (57)	135 (70)	45 (68)	32 (67)
Rectum	81 (35)	76 (34)	80 (43)	59 (30)	21 (32)	16 (33)
Site of metastases, *n* (%)					N/A	N/A
Liver only	47 (20)	34 (15)	34 (18)	47 (24)		
Liver + other	152 (66)	153 (68)	127 (68)	119 (61)		
Other only	33 (14)	37 (17)	26 (14)	28 (14)		
Number of metastases, *n* (%)					N/A	N/A
1	55 (24)	41 (18)	38 (20)	53 (27)		
2	80 (34)	84 (38)	70 (37)	57 (29)		
3+	97 (42)	99 (44)	79 (42)	83 (43)		
Missing	0 (0)	0 (0)	0 (0)	1 (<1)		
Median EuroQoL score (range)						
Health state index	0.80 (−0.48, 1.00)^a^	0.80 (−0.01, 1.00)^c^	0.80 (−0.18, 1.00)^e^	0.80 (−0.18, 1.00)	0.78 (−0.07, 1.00)^h^	0.73 (0.09, 1.00)^j^
Overall health rating	78.0 (0.0, 100.0)^b^	70.0 (6.0, 100.0)^d^	75.0 (20.0, 100.0)^f^	75.0 (20.00, 100.0)^g^	70.5 (29.0, 97.0)^i^	60.0 (9.0, 93.0)^j^

^a^ *n* = 229; ^b^ *n* = 227; ^c^ *n* = 222; ^d^ *n* = 219; ^e^ *n* = 186; ^f^ *n* = 182; ^g^ *n* = 190; ^h^ *n* = 62; ^i^ *n* = 60; ^j^ *n* = 45

*BSC* best supportive care, *ECOG* Eastern Cooperative Oncology Group, *FOLFIRI* leucovorin, 5-fluorouracil, and irinotecan, *FOLFOX* leucovorin, 5-fluorouracil, and oxaliplatin, *N/A* not available

Overall rates of compliance with QoL assessment (expressed as evaluable vs. expected assessments) were 57 % for both QoL assessments in the PRIME study, 64 % for HSI and 63 % for OHR in 181 study, and 72 % for both QoL assessments in the 408 study.

### Quality of life

Using skin toxicity definition 1, there were no statistically significant differences between the panitumumab and comparator arms in any of the three studies in terms of HSI or OHR scores from baseline to discontinuation (Table 2a). The between-group difference for HSI in the 408 study, however, was larger than the MID of 0.08, favouring BSC alone. Results were similar when using definition 2 (Table 2b) and when skin toxicity was removed from the mixed-effect model (Online Resource 1).

**Table 2:** Mixed-effect linear model of change from baseline to discontinuation of treatment in EuroQoL 5-domain health state index and overall health rating scores using skin toxicity a) definition 1 and b) definition 2

	PRIME		181		408	
	Panitumumab + FOLFOX4	FOLFOX4	Panitumumab + FOLFIRI	FOLFIRI	Panitumumab + BSC	BSC
*(a)*						
Health state index	*n* = 224	*n* = 221	*n* = 186	*n* = 194	*n* = 61	*n* = 45
Adjusted LS mean	−0.005	0.006	−0.028	0.017	−0.003	0.108
95 % confidence intervals	−0.027, 0.017	−0.022, 0.034	−0.052, −0.004	−0.049, 0.014	−0.076, 0.070	−0.103, 0.319
Difference	−0.011 (−0.042, 0.020)		−0.011 (−0.047, 0.025)		−0.111 (−0.323, 0.101)	
*p* value	0.50		0.56		0.30	
Overall health rating	*n* = 222	*n* = 218	*n* = 182	*n* = 190	*n* = 59	*n* = 45
Adjusted LS mean	−0.91	0.73	−1.19	−0.73	−0.28	−0.59
95 % confidence intervals	−2.77, 0.96	−1.67, 3.14	−2.90, 0.51	−2.89, 1.43	−4.08, 3.51	−6.35, 5.16
Difference	−1.64 (−4.26, 0.98)		−0.47 (−2.85, 1.92)		0.31 (−4.54, 5.15)	
*p* value	0.22		0.70		0.90	
*(b)*						
Health state index	*n* = 224	*n* = 221	*n* = 186	*n* = 194	*n* = 61	*n* = 44
Adjusted LS mean	0.021	0.041	−0.027	−0.016	−0.048	0.175
95 % confidence intervals	−0.014, 0.057	0.003, 0.079	−0.085, 0.032	−0.076, 0.044	−0.116, 0.019	−0.039, 0.388
Difference	−0.019 (−0.050, 0.012)		−0.011 (−0.047, 0.026)		−0.223 (−0.457, 0.010)	
*p* value	0.22		0.57		0.06	
Overall health rating	*n* = 222	*n* = 218	*n* = 182	*n* = 190	*n* = 59	*n* = 44
Adjusted LS mean	−1.65	0.40	−0.74	−0.48	−3.29	1.24
95 % confidence intervals	−4.25, 0.95	−2.36, 3.16	−4.58, 3.11	−4.41, 3.45	−7.18, 0.59	−7.00, 9.49
Difference	−2.05 (−4.59, 0.49)		−0.26 (−2.72, 2.20)		−4.54 (−14.60, 5.53)	
*p* value	0.11		0.84		0.38	

*BSC* best supportive care, *FOLFIRI* leucovorin, 5-fluorouracil, and irinotecan, *FOLFOX* leucovorin, 5-fluorouracil, and oxaliplatin, *LS* least squares

### Skin toxicity

**Fig. 1:**
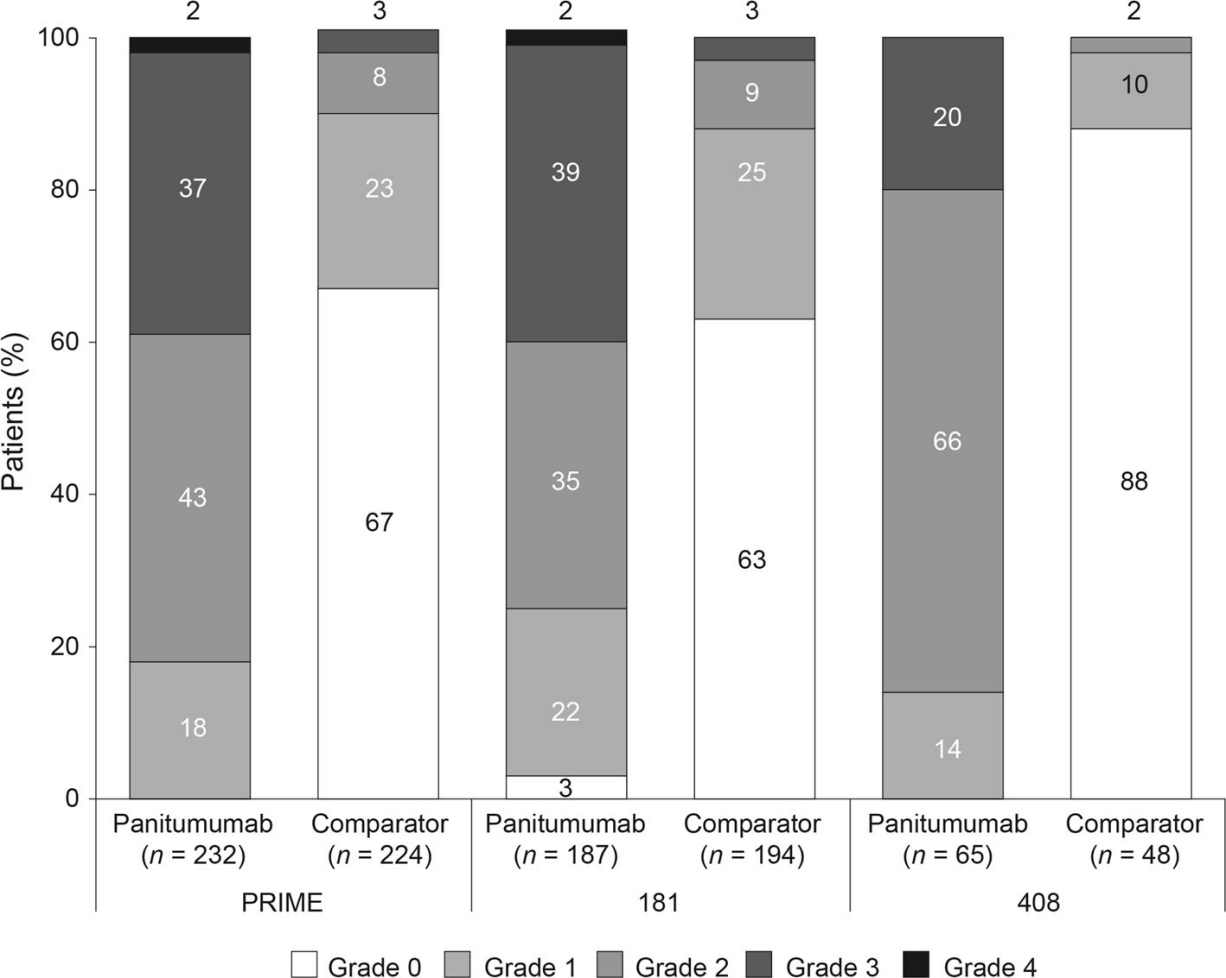
Distribution of worst skin toxicity by treatment arm in each study

The most common skin toxicities in the three studies are shown in Table 3a, with the distribution of worst skin toxicity grades by treatment in each study shown in Fig. 1. In the PRIME study, 5 % of patients overall discontinued treatment because of skin toxicities (panitumumab + FOLFOX, 8 %; FOLFOX, 1 %). In the 181 study, 4 % of patients overall discontinued treatment because of skin toxicities (panitumumab + FOLFIRI, 7 %; FOLFIRI, 0.5 %). The median number of treatment cycles received before discontinuation because of skin toxicity in the PRIME study was seven in the panitumumab + FOLFOX arm and 12 in the FOLFOX arm. The median number of treatment cycles received before discontinuation because of skin toxicity in the 181 study was five in the panitumumab + FOLFIRI arm and one in the FOLFIRI arm. The most common skin toxicities leading to discontinuation in the PRIME and 181 studies are shown in Table 3b. No patient discontinued because of skin toxicity in the 408 study.

**Table 3:** (a) Most common skin toxicities (≥10 % of patients in any treatment arm) and (b) discontinuations because of skin toxicity (>1 patient in any treatment arm)

Skin toxicity (MedDRA preferred term), *n* (%)	PRIME		181		408	
	Panitumumab + FOLFOX4 (*n* = 232)	FOLFOX4 (*n* = 224)	Panitumumab + FOLFIRI (*n* = 187)	FOLFIRI (*n* = 194)	Panitumumab + BSC (*n* = 65)	BSC (*n* = 48)
*(a)*						
Rash	131 (56)	17 (8)	105 (56)	17 (9)	12 (18)	1 (2)
Dermatitis acneiform	83 (36)	0	55 (29)	2 (1)	45 (69)	0 (0)
Pruritus	63 (27)	11 (5)	40 (21)	8 (4)	51 (78)	2 (4)
Dry skin	56 (24)	9 (4)	40 (21)	11 (6)	10 (15)	0 (0)
Skin fissures	43 (19)	1 (<1)	38 (20)	1 (1)	22 (34)	0 (0)
Erythema	42 (18)	8 (4)	27 (14)	6 (3)	49 (75)	1 (2)
Acne	36 (16)	1 (<1)	29 (16)	4 (2)	13 (20)	0 (0)
Palmar–plantar erythrodysaesthesia syndrome	24 (10)	7 (3)	20 (11)	10 (5)	1 (2)	0 (0)
Exfoliative rash	7 (3)	3 (1)	6 (3)	0 (0)	20 (31)	0 (0)

Skin toxicity (MedDRA preferred term), *n* (%)	PRIME		181	
	Panitumumab + FOLFOX4	FOLFOX4	Panitumumab + FOLFIRI	FOLFIRI
*(b)*				
Rash	10 (4)	0 (0)	6 (3)	0 (0)
Dermatitis acneiform	4 (2)	0 (0)	0 (0)	0 (0)
Acne	1 (1)	0 (0)	2 (1)	0 (0)

No patient in the 408 study discontinued because of skin toxicity

*BSC* best supportive care, *FOLFIRI* leucovorin, 5-fluorouracil, and irinotecan, *FOLFOX* leucovorin, 5-fluorouracil, and oxaliplatin, *MedDRA* Medical Dictionary for Regulatory Activities

### Analysis of quality of life by skin toxicity

Using skin toxicity definition 1, there were no statistically or clinically significant differences in QoL outcomes between patients with worst skin toxicity of grade <3 and those with grade ≥3 in any of the three studies (Table 4). In addition, there were no statistically significant differences between the panitumumab and comparator arms in subgroups of patients with worst skin toxicity grade <3 (Table 5), although the between-group difference in HSI in the 408 study (−0.112) was greater than the MID in favour of BSC. In the PRIME and 181 studies, there were no statistically significant differences between the panitumumab and comparator arms in patients with worst skin toxicity grade of ≥3, although the difference in OHR in the 181 study (−8.21) was greater than the MID in favour of FOLFIRI alone (Table 5). In the 408 study, no patient in the BSC alone arm experienced grade ≥3 skin toxicity, so no comparison was possible.

**Table 4:** Mixed-effect linear model of change from baseline to discontinuation of treatment in EuroQoL 5-domain health state index and overall health rating scores by worst skin toxicity grade (skin toxicity definition 1)

	PRIME Worst skin toxicity grade		181 Worst skin toxicity grade		408 Worst skin toxicity grade	
	<3	≥3	<3	≥3	<3	≥3
Health state index	*n* = 351	*n* = 94	*n* = 299	*n* = 81	*n* = 95	*n* = 11
Adjusted LS mean	0.007	−0.006	−0.024	−0.022	0.029	0.076
95 % confidence intervals	−0.012, 0.025	−0.040, 0.029	−0.044, −0.004	−0.059, 0.016	−0.077, 0.135	−0.078, 0.229
Difference	0.0123 (−0.026, 0.050)		−0.003 (−0.046, 0.041)		−0.047 (−0.170, 0.076)	
*p* value	0.52		0.91		0.45	
Overall health rating	*n* = 348	*n* = 92	*n* = 294	*n* = 78	*n* = 93	*n* = 11
Adjusted LS mean	−0.02	−0.15	−1.33	−0.59	−3.08	2.21
95 % confidence intervals	−1.61, 1.56	−3.03, 2.74	−2.78, 0.11	−3.20, 2.03	−6.27, 0.10	−4.87, 9.29
Difference	0.13 (−3.03, 3.29)		−0.75 (−3.65, 2.15)		−5.29 (−12.3, 1.71)	
*p* value	0.94		0.61		0.14	

*LS* least squares

**Table 5:** Mixed-effect linear model of change from baseline to discontinuation of treatment in EuroQoL 5-domain health state index and overall health rating scores by treatment group and worst skin toxicity grade in the (a) PRIME, (b) 181, and (c) 408 studies (skin toxicity definition 1)

	Worst skin toxicity grade			
	<3		≥3	
	Panitumumab + FOLFOX4	FOLFOX4	Panitumumab + FOLFOX4	FOLFOX4
*(a)*				
Health state index	*n* = 136	*n* = 215	*n* = 88	*n* = 6
Adjusted LS mean	−0.003	0.011	−0.004	0.009
95 % confidence intervals	−0.033, 0.027	−0.014, 0.036	−0.040, 0.032	−0.116, 0.134
Difference	−0.015 (−0.053, 0.024)		−0.013 (−0.140, 0.114)	
Overall health rating	*n* = 136	*n* = 212	*n* = 86	*n* = 6
Adjusted LS mean	−0.30	0.66	−1.56	2.10
95 % confidence intervals	−2.83, 2.23	−1.46, 2.78	−4.66, 1.54	−8.36, 12.57
Difference	−0.96 (−4.23, 2.32)		−3.66 (−14.30, 6.98)	

	Worst skin toxicity grade			
	<3		≥3	
	Panitumumab + FOLFIRI	FOLFIRI	Panitumumab + FOLFIRI	FOLFIRI
*(b)*				
Health state index	*n* = 111	*n* = 188	*n* = 75	*n* = 6
Adjusted LS mean	−0.019	−0.022	−0.038	0.032
95 % confidence intervals	−0.051, 0.014	−0.047, 0.004	−0.074, −0.001	−0.100, 0.164
Difference	0.003 (−0.038, 0.044)		−0.070 (−0.206, 0.066)	
Overall health rating	*n* = 110	*n* = 184	*n* = 72	*n* = 6
Adjusted LS mean	−2.10	−0.61	−1.39	6.82
95 % confidence intervals	−4.47, 0.26	−2.53, 1.32	−4.15, 1.38	−2.04, 15.68
Difference	−1.50 (−4.53, 1.53)		−8.21 (−17.26, 0.85)	

	Worst skin toxicity grade			
	<3		≥3	
	Panitumumab + BSC	BSC	Panitumumab + BSC	BSC
*(c)*				
Health state index	*n* = 50	*n* = 45	*n* = 11	*n* = 0
Adjusted LS mean	−0.027	0.084	0.026	−
95 % confidence intervals	−0.096, 0.042	−0.122, 0.291	−0.146, 0.199	−
Difference	−0.112 (−0.329, 0.106)		−	
Overall health rating	*n* = 48	*n* = 45	*n* = 11	*n* = 0
Adjusted LS mean	−2.47	−3.08	3.54	−
95 % confidence intervals	−5.72, 0.77	−14.57, 8.41	−3.51, 10.59	−
Difference	0.60 (−11.36, 12.57)		−	

*BSC* best supportive care, *FOLFIRI* leucovorin, 5-fluorouracil, and irinotecan, *FOLFOX* leucovorin, 5-fluorouracil, and oxaliplatin, *LS* least squares

**Fig. 2:**
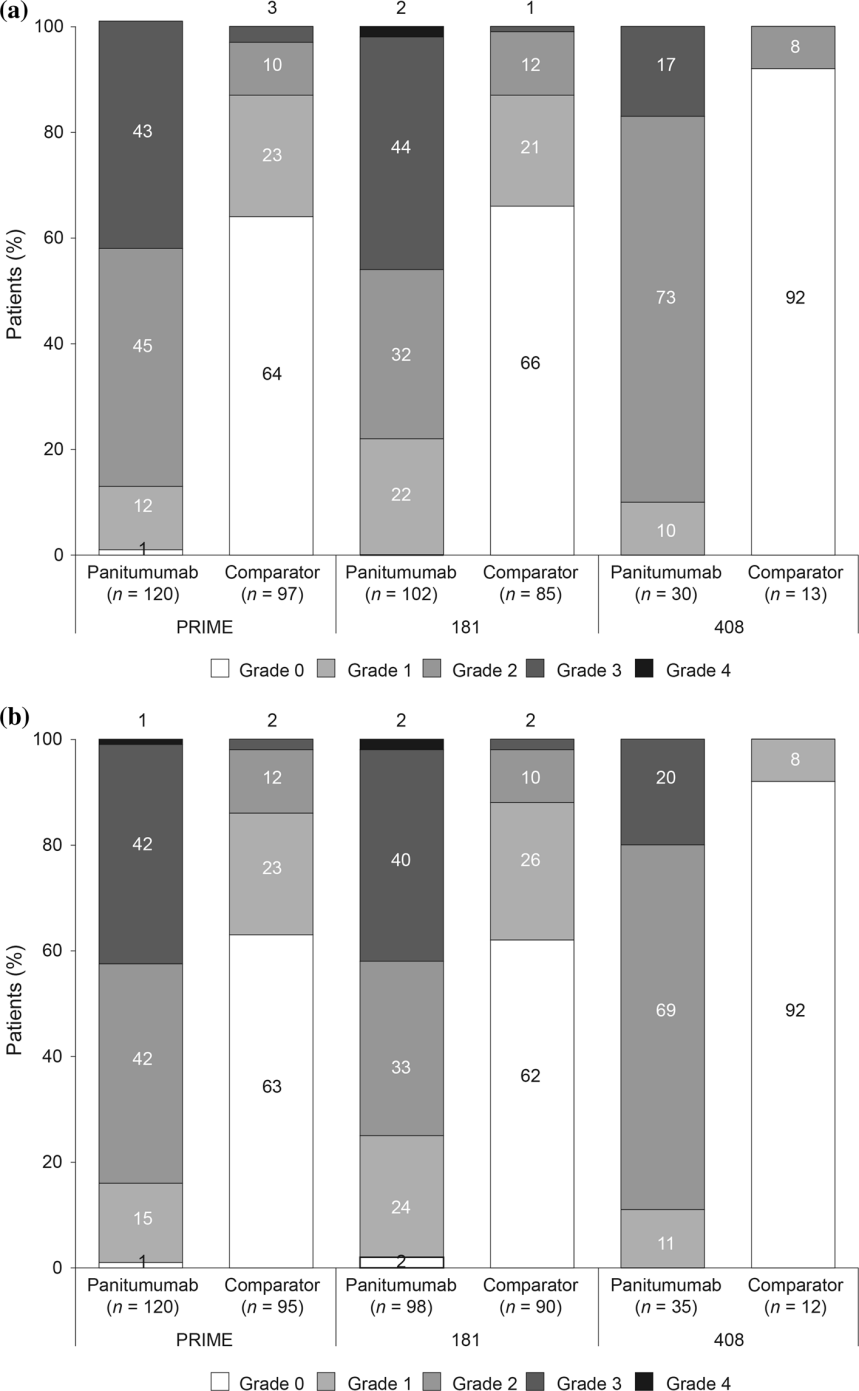
Distribution of worst skin toxicity by treatment arm in each study in patients who had clinically meaningful decreases of **a** ≥0.08 points in HSI and **b** ≥7 points in overall health rating

The distributions of worst skin toxicity grades in patients with a decrease in QoL greater than the MIDs for HSI or OHR are shown in Fig. 2.

## Discussion

While the addition of panitumumab to chemotherapy or BSC is associated with skin toxicity—as demonstrated by the distribution of worst grade skin toxicity in each treatment arm—results from these analyses show no statistically significant negative impact of panitumumab treatment on overall QoL. In addition, there was no apparent difference in distribution of worst skin toxicity grade in patients with a decrease in QoL compared with the overall population.

In first- and second-line therapy, addition of panitumumab to chemotherapy had no clinically significant impact on overall QoL. Indeed, a recent quality-adjusted time without symptoms of disease or toxicity (Q-TWiST) analysis from the PRIME study showed that panitumumab plus FOLFOX4 significantly improved quality-adjusted survival time compared with FOLFOX4 alone (20.5 vs. 18.2 months, respectively; *p* = 0.025) [22]. In later lines of therapy (408 study), the difference in HSI between the panitumumab plus BSC and BSC alone arms (−0.111 and −0.223 for skin toxicity definitions 1 and 2, respectively) was greater than the MID (0.08), although not statistically significant. This is in contrast to an earlier analysis of QoL in the *KRAS* WT population of the 408 study, which showed a small, but clinically significant benefit for panitumumab plus BSC over BSC alone on the EQ-5D HSI [23]. Furthermore, a previous Q-TWiST analysis of PFS and OS in the 408 study showed that panitumumab plus BSC significantly improved quality-adjusted survival compared with BSC alone in patients with *KRAS* WT mCRC [24]. Importantly, the small patient cohort in the 408 study should be taken into account when interpreting the results reported here. In addition, while a clinically meaningful difference between the panitumumab plus FOLFIRI and FOLFIRI alone arms was observed for patients with worst skin toxicity grade ≥3 in the 181 study, this is probably the result of the small number of patients in the FOLFIRI arm with skin toxicity of grade ≥3.

While skin toxicity might be expected to have a negative impact on QoL, there were no statistically or clinically significant differences in QoL between patients with worst skin toxicity grade <3 and those with grade ≥3 in these exploratory analyses. Furthermore, few patients discontinued as a result of skin toxicity in any of the three studies. While skin toxicity had no impact on global QoL, it is important to note that it remains a clinically relevant adverse event that requires proactive management [12].

Overall, our results are consistent with previous studies of first- and second-line EGFR inhibitor therapy in mCRC. For example, similar results were seen in an earlier analysis of QoL data from patients with *KRAS* WT mCRC in the PRIME study [25], as well as in a recent analysis of EQ-5D subscales [26]. In studies of first-line FOLFIRI combined with panitumumab or cetuximab, another EGFR inhibitor licensed for treatment of mCRC, no negative effect on QoL or social functioning has been observed during treatment [27, 28]. Similarly, a study of panitumumab added to second-line FOLFIRI showed that panitumumab improved PFS without compromising QoL [24]. A study of the psychological effects associated with cetuximab treatment in 80 consecutive patients treated at a single centre (any treatment line) showed that psychological distress was present in 41 % of patients [29]. Notably, there was a link between distress and overall QoL, but distress was not specifically linked with rash, which did not affect psychological status or social life. In another cetuximab study, patients with mCRC experienced psychological distress initially, as they processed their cancer diagnosis and dermatological side effects at the same time [30]. Later in the course of treatment, however, skin reactions no longer had a significant influence on health-related QoL, possibly because patients link the development of skin toxicities with the action of the drug and, by association, the probability of an effective treatment response.

The QoL scales used in these studies, including the EQ-5D, are measures of global QoL and are not specific for skin toxicity. The negative impact of rash-related events may therefore have been balanced by treatment-related effects, particularly in patients receiving earlier lines of therapy. For example, as noted above, patients with advanced cancer may consider skin rash to be part of their overall condition or to be a marker of efficacy [29, 30]. It is also possible that the beneficial effects of treatment—relating, for example, to symptom relief [26]—may outweigh skin-related side effects [10]. It should also be noted that rash is an expected adverse event with EGFR inhibitors, which means that physicians are able to discuss it with their patients in advance and initiate prophylactic therapy and proactive management of symptoms. The lack of a difference in QoL associated with different grades of skin toxicity suggests, however, that QoL tools specific for skin-related events are required to assess the direct effect of skin toxicity on QoL for patients receiving EGFR inhibitors.

In conclusion, the addition of panitumumab to chemotherapy in first- or second-line *RAS* WT mCRC has no negative effect on overall QoL, despite the occurrence of skin toxicity. In later lines of therapy, addition of panitumumab monotherapy to BSC was associated with no statistically significant negative effect on QoL, although some between-treatment differences were greater than the MID. In all three studies, skin toxicity of a worst grade of ≥3 appeared to have similar impact on QoL outcomes as skin toxicity of grade <3. While skin toxicity had no impact on overall QoL, it is a clinically relevant adverse event that requires proactive management. It should also be noted that the QoL scale used in these studies was a generic questionnaire, and differences may have been observed if a skin-specific QoL instrument had been used. Further research is needed, using QoL tools specific for skin-related events, to assess the direct effect of skin toxicity on QoL for patients receiving EGFR inhibitors.

## Electronic supplementary material

Below is the link to the electronic supplementary material.

Supplementary material 1 (DOCX 33 kb)

## References

[1] Yarom N, Jonker DJ (2011). The role of the epidermal growth factor receptor in the mechanism and treatment of colorectal cancer. Discovery Medicine.

[2] Keating GM (2010). Panitumumab: A review of its use in metastatic colorectal cancer. Drugs.

[3] Douillard JY, Oliner KS, Siena S, Tabernero J, Burkes R, Barugel M (2013). Panitumumab-FOLFOX4 treatment and RAS mutations in colorectal cancer. New England Journal of Medicine.

[4] Peeters, M., Oliner, K. S., Price, T. J., Cervantes, A., Sobrero, A. F., Ducreux, M., et al. (2014). Updated analysis of KRAS/NRAS and BRAF mutations in study 20050181 of panitumumab (pmab) plus FOLFIRI for second-line treatment (tx) of metastatic colorectal cancer (mCRC). *Journal of Clinical Oncology,**32*(Suppl. 5), Abstract #3568.

[5] Peeters M, Oliner KS, Parker A, Siena S, Van CE, Huang J (2013). Massively parallel tumor multigene sequencing to evaluate response to panitumumab in a randomized phase III study of metastatic colorectal cancer. Clinical Cancer Research.

[6] Patterson, S. D., Peeters, M., Siena, S., Van Cutsem, E., Humblet, Y., Van Laethem, J.-L., et al. (2013). Comprehensive analysis of *KRAS* and *NRAS* mutations as predictive biomarkers for single agent panitumumab (pmab) response in a randomized, phase III metastatic colorectal cancer (mCRC) study (20020408). *Journal of Clinical Oncology,**13*(Suppl. 15), Abstract #3617.

[7] Schwartzberg LS, Rivera F, Karthaus M, Fasola G, Canon JL, Hecht JR (2014). PEAK: A randomized, multicenter phase II study of panitumumab plus modified fluorouracil, leucovorin, and oxaliplatin (mFOLFOX6) or bevacizumab plus mFOLFOX6 in patients with previously untreated, unresectable, wild-type KRAS exon 2 metastatic colorectal cancer. Journal of Clinical Oncology.

[8] Amgen Europe B.V. (2015). *Vectibix. EPAR product information*. Breda: Amgen Europe B.V.

[9] Boyd, K. A., Briggs, A. H., Paul, J., Iveson, T., Midgely, R., Harkin, A., et al. (2011). Analysis of adverse events and quality of life data for an economic evaluation of adjuvant chemotherapy in colorectal cancer: When can we stop collecting? *Trials*, *12*(Suppl. 1), A41 (Abstract).

[10] Russi EG, Moretto F, Rampino M, Benasso M, Bacigalupo A, De Sanctis V (2015). Acute skin toxicity management in head and neck cancer patients treated with radiotherapy and chemotherapy or EGFR inhibitors: Literature review and consensus. Critical Reviews in Oncology/Hematology.

[11] American Cancer Society. Chemotherapy principles. 2015. http://www.cancer.org/acs/groups/cid/documents/webcontent/002995-pdf.pdf. Accessed 23 June 2015.

[12] Li T, Perez-Soler R (2009). Skin toxicities associated with epidermal growth factor receptor inhibitors. Targeted Oncology.

[13] Montazeri A (2009). Quality of life data as prognostic indicators of survival in cancer patients: An overview of the literature from 1982 to 2008. Health and Quality of Life Outcomes.

[14] Douillard JY, Rong A, Sidhu R (2013). RAS mutations in colorectal cancer. New England Journal of Medicine.

[15] Douillard JY, Siena S, Cassidy J, Tabernero J, Burkes R, Barugel M (2010). Randomized, phase III trial of panitumumab with infusional fluorouracil, leucovorin, and oxaliplatin (FOLFOX4) versus FOLFOX4 alone as first-line treatment in patients with previously untreated metastatic colorectal cancer: The PRIME study. Journal of Clinical Oncology.

[16] Peeters, M., Oliner, K. S., Price, T. J., Cervantes, A., Sobrero, A. F., Ducreux, M., et al. (2014). Analysis of KRAS/NRAS mutations in phase 3 study 20050181 of panitumumab (pmab) plus FOLFIRI versus FOLFIRI for second-line treatment (tx) of metastatic colorectal cancer (mCRC). *Journal of Clinical Oncology*, *32*(Suppl. 3), Abstract #LBA387.

[17] Van Cutsem E, Peeters M, Siena S, Humblet Y, Hendlisz A, Neyns B (2007). Open-label phase III trial of panitumumab plus best supportive care compared with best supportive care alone in patients with chemotherapy-refractory metastatic colorectal cancer. Journal of Clinical Oncology.

[18] Peeters M, Price TJ, Cervantes A, Sobrero AF, Ducreux M, Hotko Y (2010). Randomized phase III study of panitumumab with fluorouracil, leucovorin, and irinotecan (FOLFIRI) compared with FOLFIRI alone as second-line treatment in patients with metastatic colorectal cancer. Journal of Clinical Oncology.

[19] National Cancer Institute Cancer Therapy Evaluation Program (CTEP). Common terminology criteria for adverse events (CTCAE), version 3.0, DCTD, NCI, NIH, DHHS (2006). http://ctep.cancer.gov/protocolDevelopment/electronic_applications/docs/ctcaev3.pdf. Accessed 15 February 2016.

[20] Peeters M, Price TJ, Cervantes A, Sobrero AF, Ducreux M, Hotko Y (2014). Final results from a randomized phase 3 study of FOLFIRI ± panitumumab for second-line treatment of metastatic colorectal cancer. Annals of Oncology.

[21] Fitzmaurice GM, Laird N, Ware JH (2011). Applied longitudinal analysis.

[22] Wang, J., Hechmati, G., Dong, J., Maglinte, G. A., Barber, B., Douillard, J.-Y. (2015). Q-TWiST analysis of panitumumab plus FOLFOX4 versus FOLFOX4 alone in patients with previously untreated wild-type *RAS* metastatic colorectal cancer. *Current Medical Research and Opinion* **(Epub ahead of print)**. 10.1185/03007995.2015.1124075 26613286

[23] Odom D, Barber B, Bennett L, Peeters M, Zhao Z, Kaye J (2011). Health-related quality of life and colorectal cancer-specific symptoms in patients with chemotherapy-refractory metastatic disease treated with panitumumab. International Journal of Colorectal Disease.

[24] Wang J, Zhao Z, Barber B, Sherrill B, Peeters M, Wiezorek J (2011). A Q-TWiST analysis comparing panitumumab plus best supportive care (BSC) with BSC alone in patients with wild-type KRAS metastatic colorectal cancer. British Journal of Cancer.

[25] Bennett L, Zhao Z, Barber B, Zhou X, Peeters M, Zhang J (2011). Health-related quality of life in patients with metastatic colorectal cancer treated with panitumumab in first- or second-line treatment. British Journal of Cancer.

[26] Siena S, Tabernero J, Bodoky G, Cunningham D, Rivera F, Ruff P (2016). Quality of life during first−line FOLFOX4 ± panitumumab in RAS wild−type metastatic colorectal carcinoma: Results from a randomised controlled trial. ESMO Open.

[27] Thaler J, Karthaus M, Mineur L, Greil R, Letocha H, Hofheinz R (2012). Skin toxicity and quality of life in patients with metastatic colorectal cancer during first-line panitumumab plus FOLFIRI treatment in a single-arm phase II study. BioMed Central Cancer.

[28] Láng I, Köhne CH, Folprecht G, Rougier P, Curran D, Hitre E (2013). Quality of life analysis in patients with KRAS wild-type metastatic colorectal cancer treated first-line with cetuximab plus irinotecan, fluorouracil and leucovorin. European Journal of Cancer.

[29] Romito F, Giuliani F, Cormio C, Tulipani C, Mattioli V, Colucci G (2010). Psychological effects of cetuximab-induced cutaneous rash in advanced colorectal cancer patients. Supportive Care in Cancer.

[30] Unger K, Niehammer U, Hahn A, Goerdt S, Schumann M, Thum S (2013). Treatment of metastatic colorectal cancer with cetuximab: Influence on the quality of life. Zeitschrift für Gastroenterologie.

