# Supplementary material for: Skin toxicity and quality of life during treatment with panitumumab for RAS wild-type metastatic colorectal carcinoma: results from three randomised clinical trials
Source: Qual Life Res. 2016 Apr 15;25(10):2645–56. doi: 10.1007/s11136-016-1288-4 (PMC5010834; doi:10.1007/s11136-016-1288-4)
Supplement: Supplementary file 1 — Supplementary material 1 (DOCX 33 kb) [file 11136_2016_1288_MOESM1_ESM.docx]

Skin toxicity and quality of life during treatment with panitumumab for *RAS* wild-type metastatic colorectal carcinoma: results from three randomized clinical trials

**Quality of Life Research**

Reija Koukakis • Francesca Gatta • Guy Hechmati • Salvatore Siena

**Corresponding author**: Reija Koukakis, Amgen Ltd, 1 Uxbridge Business Park, Sanderson Road, Uxbridge UB8 1DH, Middlesex, UK. Tel: +44 (0)1895 525542; Fax: +44 (0)1895 525104; Email: [reijak@amgen.com](mailto:reijak@amgen.com)

**Supplemental Table 1** Mixed-effect piecewise linear model of change from baseline to discontinuation of treatment in EuroQol 5-domain health state index and overall health rating score, excluding skin toxicity as a covariate

|  | PRIME | | 181 | | 408 | |
| --- | --- | --- | --- | --- | --- | --- |
|  | Panitumumab + FOLFOX4 | FOLFOX4 | Panitumumab + FOLFIRI | FOLFIRI | Panitumumab + BSC | BSC |
| Health state index | *n* = 224 | *n* = 221 | *n* = 186 | *n* = 194 | *n* = 61 | *n* = 45 |
| Adjusted LS mean | –0.002 | 0.012 | –0.028 | –0.019 | –0.019 | 0.084 |
| 95% confidence intervals | –0.024, 0.019 | –0.010, 0.034 | –0.052, –0.005 | –0.042, 0.005 | –0.079, 0.042 | –0.117, 0.286 |
| Difference | –0.015 (–0.043, 0.013) | | –0.010 (–0.041, 0.022) | | –0.103 (–0.313, 0.107) | |
| *p*-value | 0.29 | | 0.55 | | 0.33 | |
| Overall health rating | *n* = 222 | *n* = 218 | *n* = 182 | *n* = 190 | *n* = 59 | *n* = 45 |
| Adjusted LS mean | –0.89 | 0.80 | –1.27 | –1.08 | –2.02 | –3.17 |
| 95% confidence intervals | –2.72, 0.94 | –1.09, 2.68 | –2.95, 0.42 | –2.76, 0.60 | –5.07, 1.03 | –7.80, 1.47 |
| Difference | –1.69 (–4.03, 0.66) | | –0.19 (–2.31, 1.93) | | 1.15 (–3.60, 5.89) | |
| *p*-value | 0.16 | | 0.86 | | 0.63 | |

*BSC* best supportive care, *FOLFIRI* leucovorin, 5 flurouracil, and irinotecan, *FOLFOX* leucovorin, 5-flurouracil, and oxaliplatin, *LS* least squares
